# The effect of temperature and nitrogen source modulation on *Pseudomonas fluorescens* AQP671 ice recrystallization inhibition activity

**DOI:** 10.1371/journal.pone.0333261

**Published:** 2025-09-25

**Authors:** Õnnela Luhila, Ildar Nisamedtinov, Toomas Paalme, Katrin Laos, Allan Olspert

**Affiliations:** 1 Department of Chemistry and Biotechnology, School of Science, Tallinn University of Technology, Tallinn, Estonia; 2 Competence Center of Food and Fermentation Technologies, Tallinn Estonia; 3 R&D, Lallemand Incorporated, Montreal, Canada; East China Normal University, CHINA

## Abstract

Cold-adapted organisms have developed many different mechanisms to protect themselves from freezing temperatures. One of these mechanisms is ice recrystallization inhibition (IRI) activity. IRI refers to the ability of certain proteins and compounds to prevent the growth of ice crystals during freeze–thaw processes. The IRI activity of *Pseudomonas fluorescens* AQP 671 culture media was evaluated in relation to various nitrogen sources, incubation temperatures and pH conditions. The highest IRI activities were achieved with amino acids L-asparagine, L-proline, or L-valine, particularly under prolonged low-temperature cultivation. A rapid increase in IRI activity was observed during the first 24 hours, in response to cold shock, correlating with cell density. The activity was detected at temperatures below 15 °C, with the highest IRI activities achieved at 5–10 °C. The optimal pH range for high IRI activity was pH 6–8, and it was negatively affected by low (2–4) and high (10–12) pH values. These findings highlight the importance of both environmental conditions and nutrient composition in the expression of IRI activity in *Pseudomonas fluorescens* AQP671 culture media.

## 1. Introduction

In cold-adapted organisms, ice-binding proteins (IBPs) have emerged as pivotal biomolecules safeguarding cellular structural integrity and viability [1]. These proteins exhibit unique interactions with ice surfaces, manifesting in two distinct properties: thermal hysteresis (TH) signifying the variance between melting and freezing points, and ice-recrystallization inhibition (IRI), i.e., the inhibition of growth of larger ice crystals at the expense of smaller ones [2]. Despite their shared role in enhancing freeze-thaw tolerance of cellular membranes and structures, TH and IRI are non-correlated phenomena [2,3].

IBPs, which vary in terms of size, structure, and activity levels, have been identified across various organisms, including fish, insects, plants, algae, fungi, and microorganisms [1,4,5]. Despite these variations, all these proteins share a common trait of exhibiting cryoprotective properties [1,2,6–9].

Microbial IBPs show a particular potential for biotechnological applications due to their relatively low cost and ease of production compared to plant or animal IBPs [10]. Most research on the practical applications of bacterial IBPs focuses on ice nucleating proteins (INPs), which have been used to enhance frozen food quality (e.g., surimi, milk, and starch solutions) and for artificial snow production [11–14]. Antifreeze-active IBPs have shown promise in crop preservation via biofertilizer enrichment and in preserving frozen foods [15,16]. For example, psychrophilic yeast, *Glaciozyma martini*, was recently found to produce an extracellular, glycosylated 27 kDa ice-binding protein with IRI activity that showed cryoprotective effects in frozen fruit and vegetable preservation as well as in the cell survival of *Saccharomyces cerevisiae* after freezing [10].

Studies suggest that nutrient composition can significantly modulate ice nucleation and thermal hysteresis activities of bacterial IBPs [17–19]. Previously isolated at the coast of Ross Island, Antarctica, a strain of *Pseudomonas fluorescens* (KUAF-68) has been noted for producing two distinct IBPs, one of them (MW 80kDa) displaying low thermal hysteresis values that could be increased in the media by the addition of L-asparagine and the other one (MW 600kDa) exhibiting ice nucleation activity that could be increased in the media by the addition of glycine [17]. In *Pseudomonas fluorescens* strain MACK-4, INP activity was reduced in the presence of inorganic nitrogen sources, suggesting that these compounds suppress IBP production. Independent experiments further indicate that the source of nitrogen exerts a stronger influence than the source of carbon on both cellular growth and IBP synthesis. Among organic nitrogen sources such as yeast extract, peptone, and tryptone, no significant differences were observed in their ability to promote IBP production [19]. In addition, supplementation of the growth medium with L-serine and L-alanine has been reported to enhance INP activity in *Pantoea ananatis* [17,18]. Despite these studies, comprehensive research on the ice recrystallization inhibition (IRI) activity of bacterial IBPs and the impact of environmental parameters on the functionality and expression of these proteins is still rather limited to date.

This study focuses on another strain of *Pseudomonas fluorescens* (AQP671) sourced from Ganavan Bay, North Scotland, which appears to produce at least two distinct IBPs based on experimental observations. The IBPs are characterized by ice recrystallization inhibition activity. Our study aims to describe the interplay of temperature, pH, and nitrogen source composition in the growth medium on the expression of IRI activity, with potential biotechnological significance.

## 2. Materials and methods

### Preparation and cultivation of *P. fluorescens* under variable environmental conditions

*Pseudomonas fluorescens* strain AQP671, isolated from Ganavan Bay, Scotland (UK), was provided by Lallemand Inc. The bacteria were maintained at 30 °C on Lysogeny Broth Agar (LB, Lab M) for 24 h and cultivated at 30 °C in mineral medium (pH 7) containing MgSO₄ (1.5 g/L) and KH₂PO₄ (1.5 g/L), adding glycerol (20 g/L) as the carbon source, and NH₄Cl (3 g/L) as the nitrogen source. Cultivation was carried out on a shaker (Innova 43 Incubator Shaker Series) at 180 rpm, followed by growth in a 7 L bioreactor (Biobench, Applikon, The Netherlands). In the bioreactor, the pH was controlled at 7.0 using titration with 5M NaOH and dissolved oxygen concentration pO2 > 10% of air saturation by adjusting the aeration rate and stirrer speed. The bioreactor was inoculated (1:9, v/v) with the 48-h shake flask-grown pre-culture and grown until glycerol was almost depleted (~40 h). Then, for initiation of IBP production, the temperature was decreased to 5 °C, and glycerol (20 g/L) and NH4Cl (3 g/L) or yeast extract (Lab M, 10 g/L) were added. The incubation length at 5 °C was 32 h for the NH_4_Cl experiment and 100 h for the experiment using yeast extract as a nitrogen source.

To study the impact of individual amino acids, a portion of the culture was also withdrawn prior to the temperature shift-down and used to inoculate shake flasks (1:9, v/v) containing 20 mL of mineral medium with glycerol (20 g/L) and a single amino acid added as the sole nitrogen source. Each flask contained only one of the following amino acids: L-alanine, L-arginine, L-asparagine, L-glutamine, L-isoleucine, L-methionine, L-proline, L-serine, L-threonine, or L-valine, added in a concentration that corresponded to 1 g N/L. These results were compared to those obtained using mineral medium supplemented with either yeast extract or ammonium chloride as the nitrogen source. The nitrogen content of the yeast extract was estimated based on the total amino acid content provided by the manufacturer. The total incubation time at 5 °C was 168 h.

Similar experiments were carried out to study the effect of temperature in the presence of L-asparagine as the nitrogen source. The temperature values of 5, 10, 15, and 20 °C were selected to represent a biologically relevant range spanning from cold stress conditions, which are known to induce ice-binding protein production, up to moderate temperatures favorable for bacterial growth. Samples were collected at 24-hour intervals for a total of 96 h.

To evaluate the effect of pH on ice recrystallization inhibition activity, the pH of the culture supernatant obtained after 24 h of incubation at 5 °C was adjusted to 2, 4, 6, 8, 10, and 12 covering the physiological pH range relevant to *Pseudomonas fluorescens’* natural environment, as well as extreme pH values to assess protein stability and activity under stress conditions.

### Analytical methods

Optical density (OD) was used to estimate the biomass concentration, expressed as grams of dry weight per liter (X* *=* *0.375 gDw/L * OD). The concentration of glycerol (using a SunChrom HPLC system equipped with a Rezex™ ROA-Organic Acid H⁺ (8%) column (Phenomenex) and both UV-Vis and refractive index (RI) detectors), extracellular protein (using Pierce™ modified Lowry assay kit, Thermo Fischer Scientific) and ice recrystallization inhibition (IRI) activity were measured in the culture supernatant after removing the biomass by centrifugation at 13 000 rpm for 1 min (Biofuge Pico, Heraeus, Hanau, Germany).

**Fig 1:**
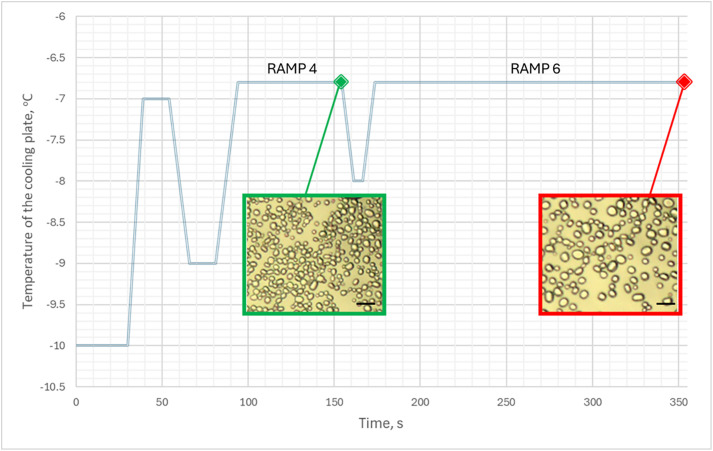
Temperature profile set points of the cooling plate for ice recrystallization inhibition (IRI) activity assessment. Images are taken at time points 154 and 354 s, highlighted in green and red accordingly. The microscope images shown are for the 35% sucrose solution at −6.8 °C.


IRI activity was measured using the modified “sucrose sandwich” assay with some modifications, as previously described [20]. 25 μl of supernatant of *P. fluorescens* AQP671 culture media was mixed with a 70% sucrose solution (1:1, v/v ratio), and 3 µl of the solution was placed on a glass microscope slide, covered with a cover slip, and the edges were sealed with silicone oil. The microscope slide was then flash-frozen in liquid nitrogen and placed on a cooling stage (Linkam PE 120, UK) of a microscope (Nikon Eclipse E 200, Japan). The temperature for the cooling stage was set to be close to the ice crystal melting point of 35% sucrose solution (T = −6.8 °C) and set to change according to the preset profile (Fig 1). The images of ice crystals were obtained using the Motic Images 3.0 program at the end of temperature ramps 4 and 6 (highlighted in green and red, respectively). Images were analyzed using the Image J image analysis tool, and the ice crystal growth rates were calculated using the Ostwald law equation [21] (Eq. 1).

$$k=\left(r^{\text{3}}-r_{\text{0}}^{\text{3}}\right)/t$$ (1)

where,

*k* – ice crystal growth rate, µm^3^/min

*r* – average ice crystal radius at the end of temperature ramp 6, µm

*r*_*0*_ – average ice crystal radius at the end of temperature ramp 4, µm

*t* – time, min.

**Fig 2:**
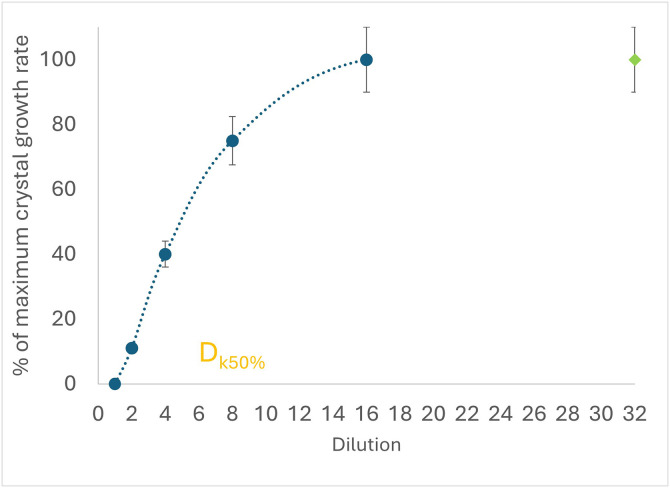
Graphical assessment of ice recrystallization inhibition (IRI) activity. Data represents mean values with standard deviation error bars (n = 3). The IRI activity is expressed as D_k50%_, calculated from the quadratic polynomial trend line (blue dotted line). The green marker at 32 × dilution indicates a plateau in ice crystal growth rates across the dilution series.

The dilution *D*_*k50%*_ of the sample, resulting in a 2-fold decrease of the maximum crystal growth speed *k,* was utilized to express the ice recrystallization inhibition activity (IRI). The dilution *D*_*k50%*_ was determined by preparing a series of 2-fold dilutions of the sample. The first dilution, 1:1 (v/v) of the sample, was made with a 70% sucrose solution, and the following 1:1 (v/v) with a 35% sucrose solution to maintain the 35% (m/v) sucrose concentration. Dilutions were made until the original sample was diluted up to 128 times (Fig 2).

The dilution D at which the ice crystal growth rate *k* reached half of the maximum speed (*D*_*k50%*_) was calculated by solving the quadratic equation (Eq. 2) corresponding to the second-order polynomial trendline of the dilution graph as follows:


$$y\;=\;aD_{k\text{5}\text{0}\%}^{\text{2}}\;+\;bD_{k\text{5}\text{0}\%}\;+\;c$$

y=50 

$$D_{k\text{5}\text{0}\%}=\frac{-b\pm \sqrt{b^{\text{2}}-\text{4}a(c-\text{5}\text{0})}}{\text{2}a}$$ (2)

where,

a, b, c – quadric, linear and constant term

y – ice crystal growth rate relative to the maximum growth rate, %

Dk_50%_ - dilution at the growth rate corresponding to 50% of the maximum growth rate

This point was defined as the *D*_*k50*%_ inhibition point, corresponding to IRI activity. All samples were measured in triplicate.

### Statistical analysis

Statistical analysis (ANOVA, two-tailed paired t-test and Pearson’s Correlation Coefficient) for the results was done with Excel Data Analysis tools. Results are presented with 95% confidence intervals. A *p*-value < 0.05 was considered statistically significant.

### Compliance with ethics requirements

This article does not contain any studies with human or animal subjects.

## 3. Results and discussion

### Expression of IRI activity in *Pseudomonas fluorescens* AQP671 culture

**Fig 3:**
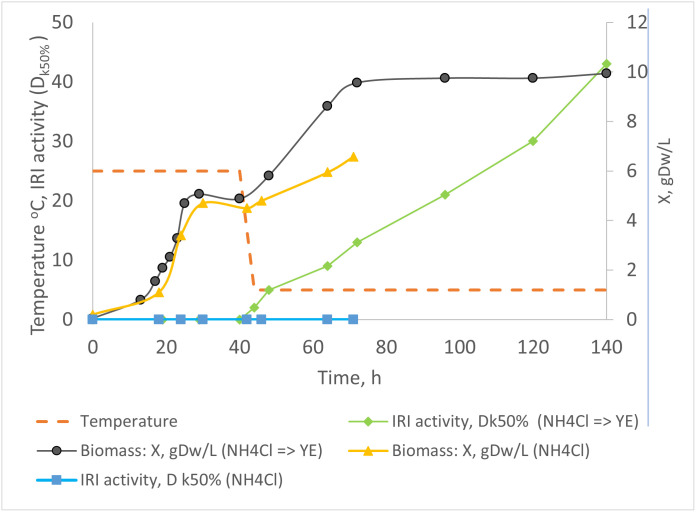
Comparison of two experiments assessing the growth and ice recrystallization inhibition (IRI) activity of Pseudomonas fluorescens AQP671 cultivated with different nitrogen sources following a temperature shift-down to 5 °C. In Experiment 1, ammonium chloride (NH₄Cl) was used as the nitrogen source throughout cultivation (biomass (X): yellow line; IRI activity: blue line). In Experiment 2, yeast extract was added as the nitrogen source after the temperature shift-down (biomass (X): black line; IRI activity: green line). The bioreactor temperature set point is shown by the orange line.

IRI activity during cultivation of *P. fluorescens* AQP671 on mineral media at 25 °C and after switching to 5 °C using yeast extract or NH_4_Cl as the nitrogen sources is shown in Fig 3. IRI activity of the culture media could not be detected during growth at 25 °C and after cultivation at the shift-down temperature (5 °C) when glycerol and NH_4_Cl were added. However, a rapid increase in IRI activity was observed if yeast extract was added to culture media instead of NH_4_Cl. Notably, IRI activity continued to rise throughout the 140 h incubation phase, reaching a dilution (D_Kd50%_) of 40, even after the culture had reached the stationary phase. Although this phenomenon has not previously been described for IBPs, other cold-inducible proteins, such as cold-shock proteins in *Bacillus subtilis* and *Caulobacter crescentus*, are known to be upregulated during the stationary phase [22,23].

### The effect of individual amino acids on ice recrystallization inhibition activity

**Fig 4:**
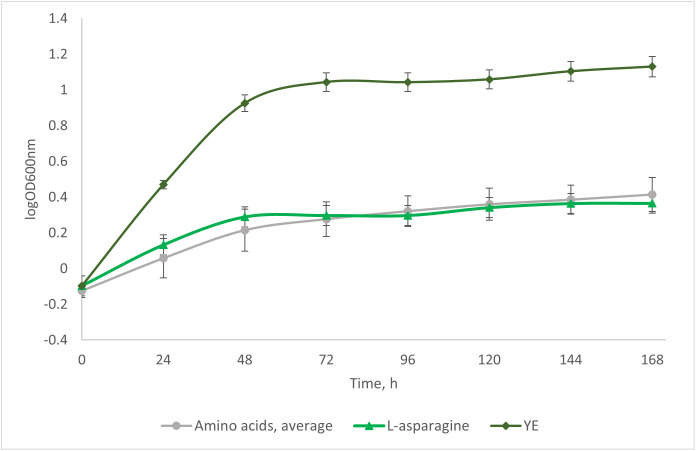
Change in the optical density during the growth of P. fluorescens AQP671 in the base medium containing a variety of amino acids or yeast extract as a nitrogen source. L-asparagine (green) is highlighted as the nitrogen source giving the highest IRI activity by the end of the experiment. Data represent mean values with standard deviation error bars (n = 3).

After reaching a biomass concentration of 10 gDw/L in the bioreactor, part of the culture was transferred into the flasks containing pre-cooled (5 °C) base media supplemented with individual organic nitrogen sources: L-alanine (A), L-arginine (R), L-asparagine (N), L-glutamine (Q), L-isoleucine (I), L-methionine (M), L-proline (P), L-serine (S), L-threonine (T), and L-valine (V), or yeast extract (YE) as a control, and incubated on shaker at 5 °C for 168 h. The biomass growth of individual nitrogen sources during the induction of IBP production by temperature shift-down is shown in Fig 4.

Despite the equal nitrogen concentration (1 g N/L) supplied with different amino acids, the growth of *P. fluorescens* AQP671 (5 gDW/L) was most pronounced in the media containing yeast extract. This can be explained by a good balance of nutrients in yeast extract [24]. In addition to various nitrogen sources, yeast extract provides sugars, vitamins, and trace elements, which can support cellular metabolism and may contribute to the increased biomass yield. Thus, the growth-promoting effect of yeast extract cannot be attributed solely to its nitrogen content [24,25]. The culture media containing a single amino acid reached much lower biomass concentrations, ranging from 0.7 gDW/L (L-threonine) to 1.5 gDW/L (L-alanine).

**Fig 5:**
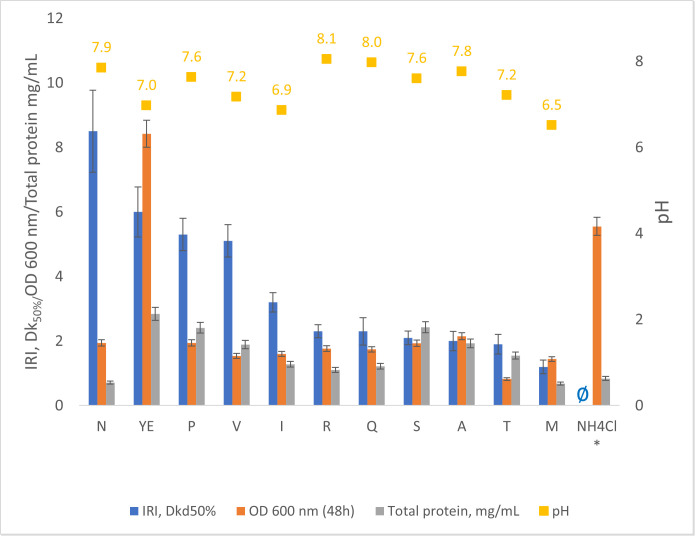
The effect of nitrogen source (L-asparagine (N), yeast extract (YE), L-proline (P), L-valine (V), L-isoleucine (I), L-arginine (R), L-glutamine (Q), L-serine (S), L-alanine (A), L-threonine (T), L-methionine (M) on P. fluorescens AQP671 IRI activity (blue bars) after 48 h incubation at 5 °C. Orange bars – the biomass concentration (OD 600 nm) at 48 h, grey bars – total protein concentration in the culture media at 168 h of incubation, yellow boxes - pH of the culture media after 168 h of incubation. Data represent mean values with standard deviation error bars (n = 3). The full data for each time point can be found in the supporting materials, S1 Table. * As a comparison, NH4Cl results correspond to the results from the experiment in the bioreactor at 71 h, shown in Fig 3, ∅ – no activity detected.

The impact of different nitrogen sources on the expression of IRI activity and total extracellular protein synthesis at 5 °C after 168 h of incubation is shown in Fig 5.

The highest IRI activity, *D*_*k50%,*_ of the culture media was achieved with L-asparagine, followed by yeast extract, L-proline, and L-valine. The other amino acids resulted in lower IRI activity. When comparing IRI activity relative to total protein content in the cultivation media, L-asparagine resulted in significantly higher specific activity than the other nitrogen sources tested.

**Fig 6:**
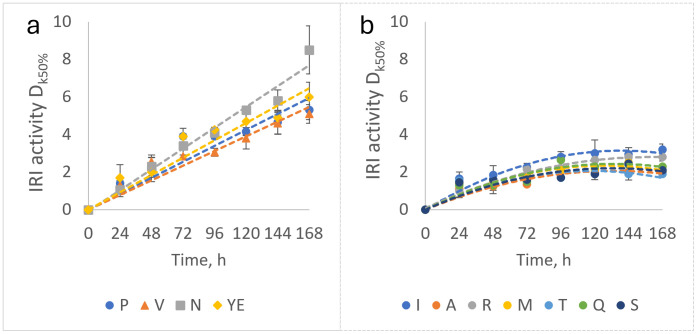
The change in ice recrystallization inhibition activity in Pseudomonas fluorescens AQP671 culture supplemented with different nitrogen sources during incubation at 5 °C on shaker, 180 rpm. a – high activity group: L-proline (P), L-valine (V), L-asparagine (N) and yeast extract (YE), b – low activity group: L-isoleucine (I), L-alanine (A), L-arginine (R) and L-methionine (M), L-threonine (T), L-glutamine (Q), L-serine (S). Data represent mean values with standard deviation error bars (n = 3).

The change in IRI activity over time can be seen in Fig 6. All the nitrogen sources showed a rapid increase in IRI activity for the first 24 h in response to the decrease in temperature.

According to the observed changes in IRI activity, the nitrogen sources were grouped into two categories. The first was the high-activity group, comprising L-asparagine, yeast extract, L-proline, and L-valine (Fig 6a), where IRI activity increased linearly (R^2^ > 0.9) throughout the entire experiment. An increase in ice recrystallization inhibition could be observed in this group during prolonged incubation at 5 °C, despite the cessation of biomass production (Fig 4).

The second group was the moderate to low-activity group, which included L-isoleucine, L-alanine, L-arginine, L-methionine, L-threonine, L-glutamine, and L-serine, with activity increasing at a much slower rate and reaching a plateau with the stop of growth (Fig 4) around D_k50%_ values of 2–3 (Fig 6b).

The measured IRI activities were very strongly in positive correlation with the cell densities measured in the culture media at different sampling points for most nitrogen sources (Pearson correlation coefficient (PCC) > 0.9), excluding L-asparagine (PCC = 0.87), L-glutamine (PCC = 0.83), and L-threonine (PCC = 0.64) which showed weaker association between cell density and IRI activity.

It can be assumed that organic nitrogen sources are required for IRI activity, as IRI activity could not be seen in the cold-induced culture when NH_4_Cl was used as the sole nitrogen source. This correlates with previous literature, where it was shown that the INP activity of *Pseudomonas fluorescens* MACK-4 IBPs was lowered in the presence of inorganic nitrogen [19]. Unfortunately, the IRI activities could not be directly related to IBP concentrations on a protein basis, as specific activity is unknown. It is worthwhile to note that similar results have been found when assessing the thermal hysteresis activity of *P. fluorescens* strain KUAF-68, which showed an almost two-fold increase in activity when L-asparagine was supplemented into the growth media at a concentration of 0.025% (w/v N) [17].

The mechanisms behind the increased IRI activity observed in the presence of particular amino acids have not been researched. However, it has been shown that asparagine/aspartate, glutamine/glutamate, arginine and serine were the amino acids most abundantly consumed throughout the growth phases for *Pseudomonas putida* [26]. Additionally, *Pseudomonas* species are known to produce enzymes facilitating the nitrogen assimilation from L-asparagine, making it an easily available nitrogen source [27]. In particular, L-asparaginase from *Pseudomonas fluorescens* has been shown to have high affinity to L-asparagine while having low affinity to glutamine [28]. Additionally, amino acids, compared to inorganic nitrogen sources, use different metabolic pathways for their assimilation, which may lead to amino-acid-specific gene regulation needed for IBP production [29,30].

### The effect of pH on ice recrystallization inhibition activity of supernatant

**Fig 7:**
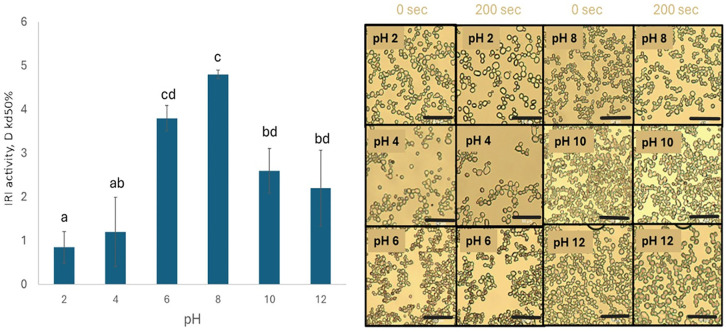
Ice recrystallization inhibition activity in P. fluorescens AQP671 culture supernatant. Data represents mean values with standard deviation error bars (n = 3). Lowercase letters indicate significant differences between samples according to the t-test values (p < 0.05) (left). Microscopic images of the ice crystals in the culture supernatant (2x diluted) at different pH values. The scale vars are 50 μm (right).

The effect of pH on IRI activity was assessed by growing bioreactor-derived precultures in 5 °C flasks containing 1 g N/L L-asparagine as nitrogen source until biomass concentration reached 1 g DW/L. The pH of the culture supernatant was then adjusted to 2, 4, 6, 8, 10, and 12 and analyzed almost immediately for ice-binding protein activity. The IRI activity of the cold-induced *P. fluorescens* AQP671 culture supernatant at different pH values is shown in Fig 7. The activity was most prominent at pH values 6 and 8 and decreased rapidly at acidic (pH 2 and pH 4) and moderately at alkaline (pH 10 and pH 12) conditions.

These results differ to some extent from previous literature data, which reported that fish and insect antifreeze proteins with both thermal hysteresis and ice recrystallization inhibition activities exhibit little to no pH dependence [31–34]. By contrast, type III antifreeze proteins and spruce budworm ice-binding proteins showed a slight decrease in absolute activity under low pH conditions, similarly to the IBPs investigated in this study [35]. This decrease was attributed to alterations in the secondary structure propensities of some protonated residues of IBPs, leading to a reduction in the total number of proximal water molecules and disrupting the water solvation structure at the basal and ice-binding interfaces of IBPs [35,36].

*Marinomonas primoryensis* IBPs (MpIBPs) exhibited the highest similarity in pH stability to *P. fluorescens* AQP671 IBPs. Thus, MpIBPs did not interact with ice at pH ≤ 4 or pH ≥ 13, at 6 ≤ pH ≤ 12 demonstrated a reduction in ice crystal grain size and at 6 ≤ pH ≤ 10 exhibited dynamic ice shaping [37]. These results indicate that the impact of pH on IBP stability is not universal but may be dependent on the species producing these IBPs and their molecular structures.

### The effect of temperature on expression of ice recrystallization inhibition activity

**Fig 8:**
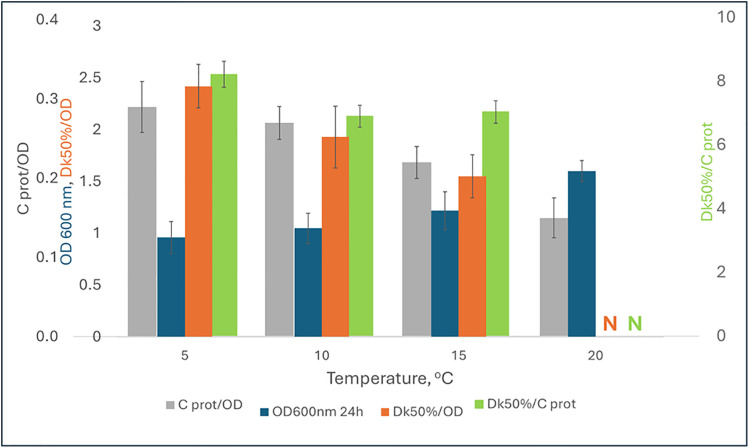
Total protein concentration normalized to cell density (gray), biomass concentration at 24 h (blue), IRI activity normalized to biomass concentration (orange) and IRI activity normalized to total protein concentration in the culture media of P. fluorescens AQP671 after incubation for 24 h at 5, 10, 15 and 20 °C. Data represent mean values with standard deviation error bars (n = 3). N – not detected.

To assess temperature effects on IRI activity, the bioreactor-grown preculture was incubated for 96 h at 5, 10, 15, or 20 °C in the medium containing L-asparagine as a nitrogen source. IRI activity in the culture supernatant was observed at temperatures of 15 °C and lower (Fig 8).

The results also show that the total protein content and IRI activity, when normalized to cell density, increased with lowering cultivation temperatures. However, similarly to the previous experiment with different amino acids, the total protein concentration in the culture media did not correlate with IRI activity, suggesting that the proteins responsible for IRI activity may constitute only a small fraction of the total proteins present. This corroborates previous findings that IRI activity can occur at as low as nano- to micromolar concentrations of IBPs [2,38].

The total protein as well as IRI activity per unit of biomass increased with the decrease of expression temperature, while the concentration of biomass decreased. IRI activity per protein content changed little, if at all (Fig 8).

The increase in IRI activity remained linear throughout the experiment at 5–10 °C. The IRI activities at 5, 10 and 15 °C were the same during the 72 h incubation (p < 0.05) and statistical differences could be seen only at the 96-hour sampling point, with the highest activity measured at 10 °C and the lowest at 15 °C (data shown in Supporting materials S2 Table).

Overall, the IBP induction temperatures determined in this work were similar to those determined for other bacterial IBPs, including *P. fluorescens* strain KUAF-68 (5 °C), *Pseudomonas putida* GR12−2 (10 °C) and *Moraxella sp* (10 °C) [17].

## 4. Conclusions

Our study demonstrates that the ice recrystallization inhibition (IRI) activity expressed by *P*. *fluorescens* AQP671 is significantly influenced by the nitrogen source, pH and incubation temperature. The highest IRI activity was observed with L-asparagine, L-proline, and L-valine, particularly under conditions of prolonged cold stress. The IRI activity was equally expressed at temperatures of 15 °C and below, while no activity was detected at temperatures above 20 °C. The activity per unit was most prominent at neutral pH values and decreased rapidly at acidic and moderately at alkaline pH values. These findings suggest that both environmental factors and nutrient composition play crucial roles in the expression of IRI activity in *Pseudomonas fluorescens* AQP671. A limitation of this study was that the IRI protein was not isolated nor sequenced, and could not be quantified in terms of mass per volume.

## Supporting information

S1 Table Ice recrystallization inhibition activity expressed as 50% dilution point, normalized to cell density and total protein content. (DOCX)

S2 Table Ice recrystallization inhibition activity (Dkd50%) in the culture medium of *P. fluorescens* AQP671 at 5, 10 and 15 °C. (DOCX)
