# Supplementary material for: The effect of temperature and nitrogen source modulation on Pseudomonas fluorescens AQP671 ice recrystallization inhibition activity
Source: PLoS One. 2025 Sep 25;20(9):e0333261. doi: 10.1371/journal.pone.0333261 (PMC12463234; doi:10.1371/journal.pone.0333261)
Supplement: S1 Table — (DOCX) [file pone.0333261.s001.docx]

***S1 Table. Ice recrystallization inhibition activity expressed as 50% dilution point, normalized to cell density and total protein content*** *(L-alanine(A), L-arginine(R), L-asparagine N), L-glutamine(Q), L-isoleucine(I), L-methionine(M), L-proline(P), L-serine(S), L-threonine(T) and L-valine(V), yeast extract(YE)) Values with the same lower-case letters within a time point do not differ significantly (α = 0.05), n = 3*

|  | **N** | | **YE** | | **P** | | | | **V** | | | | | **I** | **R** | | **Q** | | | | | **S** | | | **M** | | | **A** | | | **T** |
| --- | --- | --- | --- | --- | --- | --- | --- | --- | --- | --- | --- | --- | --- | --- | --- | --- | --- | --- | --- | --- | --- | --- | --- | --- | --- | --- | --- | --- | --- | --- | --- |
|  |  |  | |  | | |  | | | |  | | **50% dilution point** | | |  | | | | |  | | |  | |  | | |  | | |
| 0 h | ND | | ND | | ND | | | | ND | | | | | ND | ND | | ND | | | | | ND | | | ND | | | ND | | | ND |
| 24 h | 1.1 ± 0.4 ^a^ | | 1.7 ± 0.7 ^a^ | | 1.4 ± 0.1 ^a^ | | | | 1.3 ± 0.1 ^a^ | | | | | 1.6 ± 0.4 ^a^ | 1.0 ± 0.4 ^a^ | | 1.3 ± 0.5 ^a^ | | | | | 1.5 ± 0.6 ^a^ | | | 1.4 ± 0.1 ^a^ | | | 1.1 ± 0.4 ^a^ | | | 1.6 ± 0.4 ^a^ |
| 48 h | 2.3 ± 0.5 ^bcd^ | | 2.0 ± 0.3 ^bcd^ | | 2.2 ± 0.7 ^bcd^ | | | | 2.6 ± 0.2 ^bc^ | | | | | 1.9 ± 0.5 ^bd^ | 1.4 ± 0.5 ^d^ | | 1.3 ± 0.4 ^d^ | | | | | 1.6 ± 0.2 ^bd^ | | | 1.5 ± 0.1 ^bd^ | | | 1.3 ± 0.2^d^ | | | 1.6 ± 0.4 ^bd^ |
| 72 h | 3.4 ± 0.5 ^ef^ | | 3.9 ± 0.4 ^f^ | | 3.3 ± 0.1 ^ef^ | | | | 2.9 ± 0.1 ^e^ | | | | | 2.3 ± 0.2 ^g^ | 2.1 ± 0.2 ^gh^ | | 1.5 ± 0.6 ^ghi^ | | | | | 1.6 ± 0.6 ^ghi^ | | | 1.7 ± 0.1 ^h^ | | | 1.4 ± 0.1 ^i^ | | | 1.6 ± 0.4 ^ghi^ |
| 96 h | 4.1 ± 0.3 ^jk^ | | 4.3 ± 0.1 ^j^ | | 3.9 ± 0.1 ^k^ | | | | 3.1 ± 0.2 ^l^ | | | | | 2.8 ± 0.3 ^lm^ | 2.2 ± 0.6 ^lno^ | | 2.7 ± 0.1 ^mo^ | | | | | 1.7 ±0.6 ^n^ | | | 2.1 ± 0.2 ^n^ | | | 2.1 ± 0.1 ^n^ | | | 1.7 ± 0.4 ^n^ |
| 120 h | 5.3 ± 0.1 ^p^ | | 4.7 ± 0.7 ^pq^ | | 4.2 ± 0.2 ^qr^ | | | | 3.8 ± 0.6 ^qr^ | | | | | 3.0 ± 0.7 ^rst^ | 2.7 ± 0.1 ^s^ | | 2.3 ± 0.1 ^t^ | | | | | 1.9 ± 0.1 ^s^ | | | 2.2 ± 0.2 ^st^ | | | 2.0 ± 0.4 ^st^ | | | 1.9 ± 0.1 ^s^ |
| 144 h | 5.8 ± 0.6 ^u^ | | 4.9 ± 0.1 ^u^ | | 4.7 ± 0.6 ^u^ | | | | 4.6 ± 0.6 ^u^ | | | | | 3.0 ± 0.4 ^v^ | 2.8 ± 0.3 ^vw^ | | 2.4 ± 0.4 ^vwx^ | | | | | 2.5 ± 0.6 ^vwx^ | | | 2.5 ± 0.1 ^vwx^ | | | 2.0 ± 0.4 ^wx^ | | | 1.9 ± 0.4 ^x^ |
| 168 h | 8.5 ± 1.3 | | 6.0 ± 0.8 ^y^ | | 5.3 ± 0.5 ^y^ | | | | 5.1 ± 0.5 ^y^ | | | | | 3.2 ± 0.3 ^z^ | 2.8 ± 0.2 ^zž^ | | 2.3 ± 0.4 ^žš^ | | | | | 2.1 ± 0.2 ^š^ | | | 2.1 ± 0.2 ^š^ | | | 2.0 ± 0.3 ^š^ | | | 1.9 ± 0.3 ^š^ |
|  |  |  | |  | |  | | | |  | | **50% dilution point/OD** | | | | | | |  | |  | | |  | |  | | |  | | |
| 0 h | ND | | ND | | ND | | | | ND | | | | | ND | ND | | ND | | | | | ND | | | ND | | | ND | | | ND |
| 24 h | 0.8 ± 0.3 ^α^ | | 0.6 ± 0.2 ^α^ | | 1.0 ± 0.1 ^α^ | | | | 1.0 ± 0.2 ^α^ | | | | | 1.0 ± 0.3 ^α^ | 0.9 ± 0.3 ^α^ | | 1.0 ± 0.4 ^α^ | | | | | 1.0 ± 0.4 ^α^ | | | 1.1 ± 0.1 ^α^ | | | 0.8 ± 0.3 ^α^ | | | 1.4 ± 0.6 ^α^ |
| 48 h | 1.2 ± 0.3 ^af^ | | 0.2 ± 0.0 | | 1.1 ± 0.4 ^abf^ | | | | 1.7 ± 0.1 ^bc^ | | | | | 1.2 ± 0.3 ^ac^ | 0.8 ± 0.3 ^ad^ | | 0.7 ± 0.2 ^ae^ | | | | | 0.8 ± 0.1 ^a^ | | | 1.0 ± 0.1 ^a^ | | | 0.6 ± 0.1^de^ | | | 1.9 ± 0.5 ^cf^ |
| 72 h | 1.7 ± 0.2 ^gi^ | | 0.4 ± 0.0 ^h^ | | 1.5 ± 0.1 ^gi^ | | | | 1.7 ± 0.0 ^i^ | | | | | 1.4 ± 0.1 ^g^ | 1.0 ± 0.1 ^j^ | | 0.7 ± 0.3 ^hjk^ | | | | | 0.7 ± 0.3 ^hjk^ | | | 1.1 ± 0.1 ^j^ | | | 0.5 ± 0.1 ^k^ | | | 1.3 ± 0.3 ^gij^ |
| 96 h | 2.2 ± 0.1 | | 0.4 ± 0.0 ^l^ | | 1.7 ± 0.1 ^m^ | | | | 1.4 ± 0.1 ^npq^ | | | | | 1.7 ± 0.2 ^mnp^ | 1.1 ± 0.3 ^noq^ | | 1.5 ± 0.0 ^p^ | | | | | 0.6 ± 0.2 ^loq^ | | | 1.2 ± 0.1 ^nq^ | | | 0.7 ± 0.0 ^o^ | | | 1.0 ± 0.2 ^oq^ |
| 120 h | 2.4 ± 0.1 | | 0.4 ± 0.1 | | 1.6 ± 0.1 ^r^ | | | | 1.7 ± 0.3 ^rs^ | | | | | 1.5 ± 0.4 ^rt^ | 1.1 ± 0.0 ^t^ | | 1.0 ± 0.1 ^t^ | | | | | 0.6 ±0.0 ^uw^ | | | 1.2 ± 0.1 ^stu^ | | | 0.6 ± 0.1 ^w^ | | | 1.1 ± 0.1 ^tu^ |
| 144 h | 2.5 ± 0.2 ^x^ | | 0.4 ± 0.0 | | 1.5 ± 0.2 ^y^ | | | | 2.1 ± 0.3 ^x^ | | | | | 1.4 ± 0.2 ^yz^ | 1.2 ± 0.1 ^yž^ | | 1.0 ± 0.2 ^zž^ | | | | | 0.8 ± 0.2 ^žš^ | | | 1.3 ± 0.1 ^yž^ | | | 0.6 ± 0.1 ^š^ | | | 0.9 ± 0.2 ^ž^ |
| 168 h | 3.7 ± 0.5 | | 0.4 ± 0.1 ^ü^ | | 1.5 ± 0.1 ^õ^ | | | | 2.4 ± 0.2 | | | | | 1.3 ± 0.1 ^õö^ | 1.1 ± 0.1 ^öä^ | | 1.1 ± 0.2 ^ö^ | | | | | 0.7 ± 0.1 | | | 1.0 ± 0.1 ^ä^ | | | 0.5 ± 0.1 ^ü^ | | | 1.0 ± 0.2 ^ö^ |
|  |  |  | |  | | |  | **50% dilution point/Total protein concentrations** | | | | | | | | | |  | |  | | |  | |  | |  | | |  | |
| 0 h | ND | | ND | | ND | | | | ND | | | | | ND | ND | | ND | | | | | ND | | | ND | | | ND | | | ND |
| 24 h | 2.1 ± 0.7 ^a^ | | 0.8 ± 0.3 ^b^ | | 3.7 ± 0.4 ^cd^ | | | | 4.5 ± 0.5 ^d^ | | | | | 3.9 ± 0.9 ^abcd^ | 2.6 ± 0.9 ^abc^ | | 3.2 ± 1.2 ^abcd^ | | | | | 3.4 ± 1.5 ^abcd^ | | | 3.1 ± 0.3 ^abc^ | | | 3.0 ± 1.1 ^abcd^ | | | 4.0 ± 0.9 ^abcd^ |
| 48 h | 3.7 ± 0.8 ^eh^ | | 1.2 ± 0.2 ^f^ | | 2.4 ± 0.8 ^eghij^ | | | | 3.8± 0.3 ^h^ | | | | | 2.4 ± 0.6 ^ei^ | 2.1 ± 0.8 ^efgi^ | | 1.7 ± 0.6 ^fgi^ | | | | | 2.1 ± 0.3 ^fgi^ | | | 2.4 ± 0.2 ^gi^ | | | 1.2 ± 0.2 ^fj^ | | | 3.0 ± 0.8 ^eghi^ |
| 72 h | 4.7 ± 0.7 | | 2.2 ± 0.2 ^k^ | | 2.4 ± 0.1 ^kl^ | | | | 3.1 ± 0.1 ^m^ | | | | | 2.8 ± 0.3 ^lm^ | 2.6 ± 0.3 ^klm^ | | 1.4 ± 0.6 ^kln^ | | | | | 1.4 ± 0.6 ^ko^ | | | 3.3 ± 0.3 ^m^ | | | 0.8 ± 0.1 ^no^ | | | 2.8 ± 0.7 ^klm^ |
| 96 h | 5.1 ± 0.3 | | 2.1 ± 0.0 ^p^ | | 2.2 ± 0.1 ^p^ | | | | 2.5 ± 0.2 ^qr^ | | | | | 2.8 ± 0.3 ^r^ | 2.4 ± 0.6 ^pqr^ | | 2.1 ± 0.1 ^p^ | | | | | 1.2 ± 0.4 ^st^ | | | 3.8 ± 0.4 | | | 1.1 ± 0.2 ^t^ | | | 2.2 ± 0.5 ^pqrs^ |
| 120 h | 6.8 ± 0.2 | | 2.1 ± 0.3 ^uvx^ | | 1.9 ± 0.1 ^v^ | | | | 3.0 ± 0.4 ^wx^ | | | | | 2.7 ± 0.6 ^uv^ | 3.0 ± 0.1 ^w^ | | 1.7 ± 0.1 ^uv^ | | | | | 1.3 ± 0.1 ^y^ | | | 4.1 ± 0.4 | | | 1.0 ± 0.2 ^y^ | | | 2.0 ± 0.1 ^uv^ |
| 144 h | 8.5 ± 0.8 | | 1.9 ± 0.1 ^zž^ | | 1.9 ± 0.3 ^zž^ | | | | 2.8 ± 0.3 ^š^ | | | | | 2.3 ± 0.3 ^šž^ | 3.5 ± 0.4 ^šü^ | | 1.7 ± 0.3 ^zž^ | | | | | 1.4 ± 0.4 ^zõ^ | | | 3.8 ± 0.2 ^ü^ | | | 1.0 ± 0.2 ^õ^ | | | 1.8 ± 0.4 ^zžõ^ |
| 168 h | 12.0 ± 1.8 | | 2.1 ± 0.3 ^öä^ | | 2.2 ± 0.2 ^öä^ | | | | 2.7 ± 0.3 ^ä^ | | | | | 2.5 ± 0.2 ^öä^ | 2.5 ± 0.2 ^öä^ | | 1.9 ± 0.4 ^öα^ | | | | | 0.9 ± 0.1 ^β^ | | | 3.1 ± 0.3 ^ä^ | | | 1.0 ± 0.2 ^βγ^ | | | 1.2 ± 0.2 ^αγ^ |
