# Supplementary material for: The effect of temperature and nitrogen source modulation on Pseudomonas fluorescens AQP671 ice recrystallization inhibition activity
Source: PLoS One. 2025 Sep 25;20(9):e0333261. doi: 10.1371/journal.pone.0333261 (PMC12463234; doi:10.1371/journal.pone.0333261)
Supplement: S2 Table — (DOCX) [file pone.0333261.s002.docx]

***S2 Table. Ice recrystallization inhibition activity (Dkd50%) in the culture medium of P. fluorescens AQP671 at 5, 10 and 15 °C.*** *N – not detected. Values with the same lower-case letters within a time point do not differ significantly (α = 0.05), n = 3*

| Time, h | 5 | 10 | 15 |
| --- | --- | --- | --- |
| 0 | N | N | N |
| 24 | 2.35 ± 0.21 ^a^ | 2.05 ± 0.78 ^a^ | 1.90 ± 0.42 ^a^ |
| 48 | 3.15 ± 0.64 ^b^ | 2.85 ± 0.91 ^b^ | 3.65 ± 1.20 ^b^ |
| 72 | 4.45 ± 0.50 ^c^ | 4.5 ± 0.71 ^c^ | 4.95 ± 0.50 ^c^ |
| 96 | 7.00 ± 0.79 ^d^ | 9.15 ± 1.20 ^e^ | 5.5 ± 0.57 ^f^ |
